# Evaluation of the Impact of a Chronic Total Coronary Occlusion on Ventricular Arrhythmias and Long‐Term Mortality in Patients With Ischemic Cardiomyopathy and an Implantable Cardioverter‐Defibrillator (the eCTOpy‐in‐ICD Study)

**DOI:** 10.1161/JAHA.118.008609

**Published:** 2018-05-02

**Authors:** Ivo M. van Dongen, Dilek Yilmaz, Joëlle Elias, Bimmer E. P. M. Claessen, Ronak Delewi, Reinoud E. Knops, Arthur A. M. Wilde, Lieselot van Erven, Martin J. Schalij, José P. S. Henriques

**Affiliations:** ^1^ Department of Cardiology Heart Center Academic Medical Center – University of Amsterdam The Netherlands; ^2^ Department of Cardiology Leiden University Medical Center Leiden The Netherlands

**Keywords:** chronic total coronary occlusion, implantable cardioverter‐defibrillator, ischemic cardiomyopathy, mortality, ventricular arrhythmia, Catheter Ablation and Implantable Cardioverter-Defibrillator, Coronary Artery Disease, Mortality/Survival, Arrhythmias

## Abstract

**Background:**

Previous studies report conflicting results about a higher incidence of ventricular arrhythmias in patients with a chronic total coronary occlusion (CTO). We aimed to investigate this association in a large cohort of implantable cardioverter defibrillator patients with long‐term follow‐up.

**Methods and Results:**

All consecutive patients from 1992 onwards who underwent implantable cardioverter defibrillator implantation for ischemic cardiomyopathy at the Leiden University Medical Center were evaluated. Coronary angiograms were reviewed for the presence of a CTO. The occurrence of ventricular arrhythmias and survival status at follow‐up were compared between patients with and patients without a CTO. A total of 722 patients constitute the study cohort (age 66±11 years; 84% males; 74% primary prevention, median left ventricular ejection fraction 30% [first–third quartile: 25–37], 44% received a cardiac resynchronization therapy defibrillator). At baseline, 240 patients (33%) had a CTO, and the CTOs were present for at least 44 (2–127) months. The median follow‐up duration was 4 (2–6) years. On long‐term follow‐up, CTO patients had a higher crude appropriate device therapy rate (37% versus 27%, *P*=0.010) and a lower crude survival rate (51% versus 67%, *P*<0.001) compared with patients without a CTO. Corrected for baseline characteristics including left ventricular ejection fraction, the presence of a CTO was an independent predictor for appropriate device therapy.

**Conclusions:**

The presence of a CTO in implantable cardioverter defibrillator patients was associated with more appropriate device therapy and worse prognosis at long‐term follow‐up. Further investigation is warranted regarding a potential beneficial effect of CTO revascularization on the incidence of ventricular arrhythmias.

**Clinical Perspective**

**What Is New?**

- In a large cohort of patients with an implantable cardioverter defibrillator for ischemic cardiomyopathy, the presence of a chronic total coronary occlusion is associated with higher mortality and more appropriate device therapy rates, and chronic total coronary occlusion revascularization may influence this association positively.

**What Are the Clinical Implications?**

- When clinicians encounter a patient with a chronic total coronary occlusion, with or without an implantable cardioverter defibrillator, they should be aware of a potentially higher ventricular arrhythmia occurrence in these patients, and chronic total coronary occlusion revascularization and/or implantable cardioverter defibrillator therapy should be considered carefully for improving survival and patient burden.

The presence of a chronic total coronary occlusion (CTO) has been associated with a worse prognosis compared with patients with multivessel disease (MVD) without a CTO or single‐vessel disease (SVD).^1^ This has been reported both in the setting of stable coronary artery disease and in ST‐segment elevation myocardial infarction patients, with or without cardiogenic shock.^2^, ^3^, ^4^ The underlying mechanism for this poorer prognosis is currently incompletely understood. Additionally, sudden cardiac death appears to occur more frequently in the long term in patients with a nonrevascularized CTO compared with patients with a revascularized CTO.^5^ Furthermore, in patients with ischemic systolic heart failure (ejection fraction ≤35%), the presence of a CTO has been associated with an increased 2‐year mortality.^6^

One of the hypotheses is that a CTO has a malicious influence on cardiac electrical stability, which makes patients with a CTO more prone to ventricular arrhythmias (VA),^1^, ^7^ leading to a higher mortality rate. There are limited data on the presence of a CTO and a higher incidence of VA in small implantable cardioverter‐defibrillator (ICD) patient populations, and reports are limited to short‐term follow‐up.^8^, ^9^, ^10^ The aim of the current eCTOpy‐in‐ICD (evaluation of the impact of a Chronic Total coronary Occlusion on ventricular arrhythmias and long‐term mortality in patients with ischemic cardiomyopathy and an Implantable Cardioverter‐Defibrillator) study was to investigate this association in a larger cohort of ICD patients with long‐term follow‐up, and to find variables associated with the occurrence of higher VA rates in CTO patients compared with patients without a CTO.

## Methods

The data, analytic methods, and study materials will not be made available to other researchers for purposes of reproducing the results or replicating the procedure.

### Patient Population

All consecutive patients from the year 1992 onward who underwent ICD implantation for primary or secondary prevention at the Leiden University Medical Center and had known ischemic heart disease were evaluated. Patient baseline characteristics, ICD‐related and clinical follow‐up were prospectively collected in the departmental Cardiology Information System (EPD‐vision^®^; Leiden University Medical Center, Leiden, The Netherlands). Indications for the ICD implantations were at the treating physician's discretion according to international guidelines.^11^ All data for this study have been collected during routine clinical practice. Therefore, the institutional review board approved all data collection and informed consent was waived by the Leiden University Medical Center ethics committee.

### Coronary Artery Disease Assessment

Coronary angiograms of all ICD patients were retrospectively analyzed to identify patients with 1 or more CTOs. All angiograms before the ICD implantation were considered eligible as well as all angiograms performed within the first year after the ICD implantation, unless any coronary event occurred from ICD implantation to angiogram. Preferably, the most recent coronary angiogram before the ICD implantation was used. A CTO was defined as a total coronary artery occlusion of 1 of the main arteries or large side‐branches, with thrombolysis in myocardial infarction (TIMI) 0 flow and an assumed duration of ≥3 months (based on prior angiograms/prior documented myocardial infarction in the area of the CTO). In case of the presence of a CTO, the location of the CTO was recorded in a database following a 16‐segment subdivision of the coronary artery tree.^12^ Any additional lesions were also recorded in the database, and MVD was defined as a lesion >50% in, or previous percutaneous coronary intervention (PCI) of, or coronary artery bypass grafting (CABG) of 2 or more main coronary arteries or large side‐branches (ie, diagonal branch or obtuse marginal branch).

Additionally, the quality of collaterals to the CTO was assessed using the Rentrop grade system.^13^ Of the CTO patients who underwent revascularization (either PCI or CABG), surgery reports and PCI angiograms were assessed to investigate whether or not the CTO lesion was successfully revascularized. In case of successful CTO revascularization before the ICD implantation, the patient was allocated to the non‐CTO group. In case of (re)occlusion of the CTO lesion or bypass, the patient was placed in the CTO group.

### Device Implantation, Programming, and Device Interrogation

All devices that were used were manufactured by Biotronik (Berlin, Germany), Boston Scientific (Natick, MA), Medtronic (Minneapolis, MN), or St. Jude Medical (St Paul, MN). A single‐chamber ICD, or a dual‐chamber ICD, or a Cardiac Resynchronization Therapy Defibrillator (CRT‐D) was implanted.^14^ Following implantation, sensing and pacing thresholds were tested, and a defibrillation threshold test was performed. At the start of the data collection for the registry, up until the year 2000, most devices were programmed with a single zone in which shocks were programmed to terminate VA >185 beats/min. From 2004 onward, the devices were programmed with 3 zones: VA with a frequency from 150 to 188/190 were detected in a monitoring zone and no therapy was programmed (30–32 intervals were needed for detection [NID] or 8/10 with a 2.5‐s initial delay, depending on the manufacturer).^14^ VA with a frequency >188/190 per minute were detected in the ventricular tachycardia (VT) zone, programmed with 2 to 4 bursts of antitachycardia pacing to terminate the VA, followed by shock if the VA persists (22–30 intervals needed for detection or 8/10 with a 2.5‐s initial delay, depending on the manufacturer). The final zone was programmed to detect VA >220 to 231 beats/min, in which shock was the first therapy (12–30 intervals needed for detection or 8/10 with a 1.0‐s initial delay, depending on the manufacturer).^14^ From 2008 to 2009 onward, antitachycardia pacing was programmed during charging in the last zone, depending on the manufacturer. Furthermore, supraventricular tachycardia discriminators were enabled, and atrial arrhythmia detection was set to >170 beats/min.^14^

### Follow‐Up

Periodic follow‐up visits were performed every 3 to 6 months. During follow‐up visits, patients were clinically assessed, and devices were interrogated. During this device interrogation, stored episodes were analyzed and defibrillator interventions were registered. Device therapy was classified on the basis of intracardiac electrograms and was considered appropriate only when occurring in response to VT or ventricular fibrillation (VF). All other triggers for therapy were considered inappropriate.

In the case of emigration or transmigration resulting in referral to centers far from the primary center, or when follow‐up visits were not performed for ≥12 months, follow‐up was considered incomplete. These patients were censored in the analysis at last known date of contact. In the case of heart transplantation or premature termination of ICD treatment, follow‐up was ended at the time of intervention. Patients who did not reach any end point remained at risk until the end of the study. Survival status was retrieved from regularly updated municipal civil registries. In all deceased patients, the cause of death was retrieved from hospital letters or follow‐up reports if present, and otherwise from the general practitioner.

### End Points and Definitions

The end points of interest for this study were the occurrence of appropriate device therapy (as a substitute for VA occurrence) and all‐cause mortality, hypothesizing that in the CTO group these end points would occur more often. Appropriate device therapy was defined as shock or antitachycardia pacing for VF/VT. Therapy delivered for anything other than VT or VF was defined as inappropriate. Additionally, (patient and coronary) variables associated with the occurrence of appropriate device therapy and all‐cause mortality were identified. Furthermore, we investigated the impact of a CTO on end points in patients with an ICD for primary as compared with secondary prevention, the role of collateral vessels to the CTO, and the influence of SVD and MVD with or without a CTO on outcomes. At baseline, we calculated an adjusted MADITII (Multicenter Automatic Defibrillator Implantation Trial II) risk score for all patients. The previously published MADITII risk score^15^ entails presence of New York Heart Association functional class >II, atrial fibrillation at baseline, a QRS duration of >120 ms, age >70 years, and blood urea nitrogen >26 mg/dL. Presence of any of these variables was scored as 1, and per patient a total risk score was calculated (with a maximum score of 5). Since blood urea nitrogen is not used regularly in the clinical setting in The Netherlands, we used creatinine level >1.3 μmol/L instead, which does not influence the sensitivity of the risk score as assessed by the developers of the risk score.^15^

### Statistical Analysis

Continuous variables are depicted as mean (±SD) or median (interquartile range), and comparisons between groups were made using independent *t* test or nonparametric tests, respectively. Categorical variables are depicted as frequencies (percentage of total), and comparisons between groups were made with the Fisher exact test or χ^2^ test when applicable. All‐cause mortality event rates within groups are depicted with Kaplan–Meier curves, and were compared using the log‐rank test. Kaplan–Meier estimates for 10‐year follow‐up were derived from the analyses, and depicted as the estimate rates with SE. Cox proportional hazards regression was used to assess the predictive value of variables on outcomes, after visual verification of the proportionality assumption. Appropriate variables were included after backward stepwise selection, excluding variables with a *P*>0.10. For appropriate device therapy, the left ventricular ejection fraction (LVEF) was forced into the multivariate model since it is a known strong predictor for device therapy. Overall, a *P* value of <0.05 was considered statistically significant. All analyses were performed using SPSS (Version 24; IBM Corp., Armonk, NY).

## Results

### Inclusion and Patient Population

**Figure 1:**
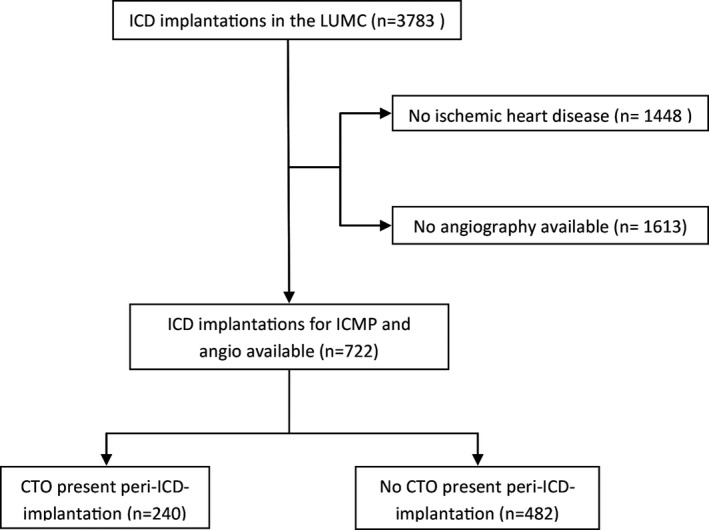
Flow diagram of patient inclusion. CTO indicates chronic total coronary occlusion; ICD, implantable cardioverter defibrillator; ICMP, ischemic cardiomyopathy; LUMC, Leiden University Medical Center.

In total, the cohort consisted of 722 patients with an ICD for ischemic cardiomyopathy (ICM) for whom an angiography was available (see Figure 1 for the flow diagram). Of these 722 patients, 275 (38%) had a CTO before the ICD implantation and 35 (13%) of these patients underwent CTO treatment (either PCI or CABG) before the ICD implantation, resulting in a total number of 240 patients (33%) with a confirmed CTO at the time of ICD implantation. Fifty‐nine of these patients (25%) had >1 CTO at baseline. The median time between date of the angiography used for CTO identification and date of ICD implantation was 1 (0.4–15) week. The median documented duration of the CTOs (age of the CTOs) was 44 (2–127) months.

The baseline patient and angiographic characteristics are shown in Tables 1 and 2. Patients in the CTO group were older, more frequently had diabetes mellitus and hypercholesterolemia, had a lower creatinine clearance, had a lower LVEF, had a longer QRS duration, and more frequently had MVD compared with patients without a CTO. Also, in CTO patients the ICD indication was more often secondary prevention. Type of device did not differ between patients with a CTO and the non‐CTO patients. The MADITII risk score showed a trend towards more CTO patients with risk scores 1 and 2, and more non‐CTO patients with a risk score of 0.

**Table 1:** Patient Baseline Characteristics

	Overall (n=722)	CTO (n=240)	Non‐CTO (n=482)	*P* Value
Age, y	66±11	67±10	65±11	0.019
Male	605 (84%)	208 (87%)	397 (82%)	0.163
Diabetes mellitus	194 (27%)	75 (31%)	119 (25%)	0.009
Hypertension	334 (46%)	115 (51%)	219 (48%)	0.466
Hypercholesterolemia	327 (45%)	133 (55%)	194 (40%)	0.001
Family history of CVD	270 (37%)	90 (38%)	180 (37%)	0.295
Family history of SCD	37 (5%)	11 (5%)	26 (5%)	0.568
Creatinine clearance	73 [52–99]	70 [50–95]	76 [55–100]	0.019
History of CABG	268 (37%)	86 (36%)	182 (38%)	0.424
ICD indication				0.031
Primary prevention	531 (74%)	164 (68%)	367 (76%)	
Secondary prevention	191 (27%)	76 (32%)	115 (24%)	
ICD type				0.383
Single‐ or dual chamber	402 (56%)	128 (53%)	274 (57%)	
CRT‐D	320 (44%)	112 (47%)	208 (43%)	
AFl/AF	218 (30%)	67 (28%)	151 (31%)	0.390
LVEF	30% [25–37]	30% [22–35]	31% [26–38]	0.001
QRS duration, ms	120 [100–150]	120 [104–156]	117 [100–150]	0.029
Medication use				
β‐Blocker	504 (70%)	158 (66%)	346 (72%)	0.103
Sotalol	79 (11%)	21 (9%)	58 (12%)	0.207
Calcium antagonist	77 (11%)	30 (13%)	47 (10%)	0.306
Amiodarone	111 (15%)	45 (19%)	66 (14%)	0.080
Digoxin	55 (8%)	17 (7%)	38 (8%)	0.767
Documented age CTO, mo	···	43.7 [2.1–127.3]	···	···
MADITII risk score^a^				0.070
0	157 (22%)	40 (17%)	117 (24%)	
1	178 (25%)	54 (23%)	124 (26%)	
2	167 (23%)	69 (29%)	98 (20%)	
3	128 (18%)	45 (19%)	83 (17%)	
4	65 (9%)	23 (10%)	42 (9%)	
5	27 (4%)	9 (4%)	18 (4%)	

AF indicates atrial fibrillation; AFl, atrial flutter; CABG, coronary artery bypass graft; CRT‐D, cardiac resynchronization therapy defibrillator; CTO, chronic total coronary occlusion; CVD, cardiovascular disease; ICD, implantable cardioverter defibrillator; LVEF, left ventricular ejection fraction; MADITII, Multicenter Automatic Defibrillator Implantation Trial II; SCD, sudden cardiac death.

a Adjusted: creatinine level >1.3 μmol/L instead of blood urea nitrogen level >26 mg/dL.

**Table 2:** Angiographic Baseline Characteristics

	Overall (n=722)	CTO (n=240)	Non‐CTO (n=482)	*P* Value
CTO vessel				···
LAD	···	68 (28%)	···	
RCx	···	48 (20%)	···	
RCA	···	124 (52%)	···	
Proximal location CTO	···	133 (55%)	···	···
Max collateral Rentrop				···
Grade 0	···	37 (15%)	···	
Grade 1	···	101 (42%)	···	
Grade 2	···	60 (25%)	···	
Grade 3	···	33 (14%)	···	
Unknown	···	9 (4%)		
Type of collateral filling				···
Bridge	···	28 (12%)	···	
Retrograde	···	145 (60%)	···	
Both	···	21 (9%)	···	
Vessel disease (VD)				<0.001
1VD	187 (26%)	34 (14%)	153 (32%)	
2VD	205 (28%)	62 (26%)	143 (30%)	
3VD	330 (46%)	144 (60%)	186 (39%)	

CTO indicates chronic coronary total occlusion; LAD, left anterior descending coronary artery; RCA, right coronary artery; RCx, ramus circumflexus coronary artery.

### Long‐Term Outcomes

On long‐term follow‐up, with a median duration of 4 (2–6) years, the overall crude cumulative event rates in this ICD population were 30% for appropriate device therapy and 39% for all‐cause mortality. Both appropriate device therapy and all‐cause mortality occurred more frequently in the CTO group, compared with the non‐CTO group (37% versus 27%, *P*=0.010, and 49% versus 33%, *P*<0.001, respectively) (Table 3).

**Table 3:** Cumulative Crude Long‐Term Event Rates

	Overall (n=722)	CTO (n=240)	Non‐CTO (n=482)	*P* Value
All‐cause mortality	279 (39%)	118 (49%)	161 (33%)	<0.001
Cause of death, n (%) of observed deaths				0.538
Tachyarrhythmic	8 (2.9%)	3 (3%)	5 (3%)	
Bradyarrhythmic	2 (1%)	1 (1%)	1 (1%)	
Heart failure	73 (26%)	26 (22%)	47 (29%)	
SCD	6 (2%)	1 (1%)	5 (3%)	
Cardiovascular	28 (10%)	13 (11%)	15 (9%)	
Appropriate device therapy	219 (30%)	88 (37%)	131 (27%)	0.010
ATP	155 (71%)	64 (73%)	91 (70%)	0.651
Shock	132 (60%)	53 (60%)	79 (60%)	1.000
Time‐to‐first appropriate device therapy in years	2.8 [1.0–5.2]	2.3 [0.9–4.4]	3.1 [1.1–5.6]	0.019
VT ablation	12 (2%)	5 (2%)	7 (2%)	0.555

ATP indicates antitachypacing; CTO, chronic total coronary occlusion; SCD, sudden cardiac death; VT, ventricular tachycardia.

**Figure 2:**
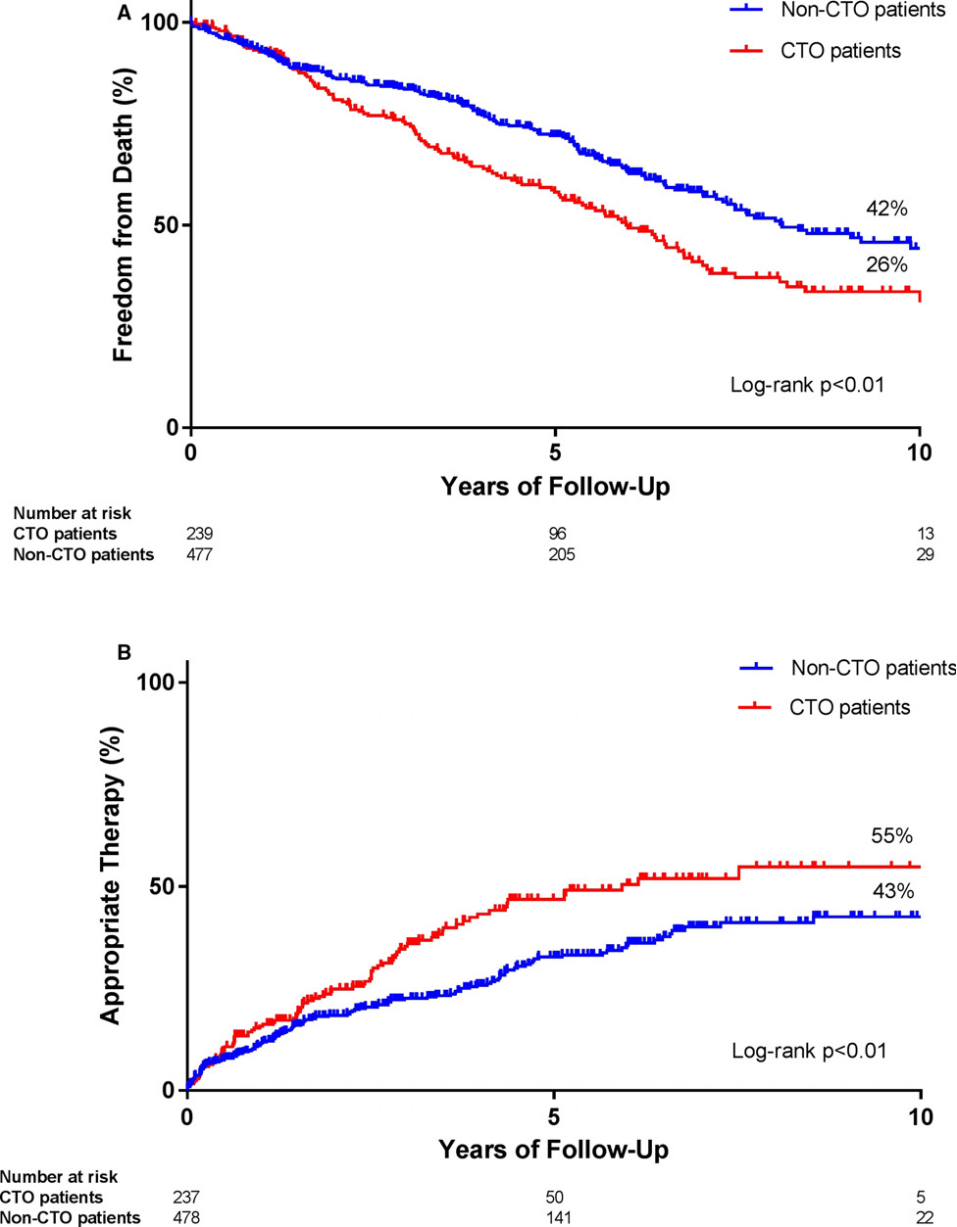
Kaplan–Meier curves for freedom from death (A) and appropriate device therapy (B), comparing patients with and without a CTO. CTO indicates chronic total coronary occlusion.

Figure 2 shows the Kaplan–Meier curves for appropriate device therapy and survival, comparing patients with and without a CTO. The 10‐year appropriate device therapy Kaplan–Meier estimates were 55 (SE 5)% for patients with a CTO and 43 (SE 4)% for patients without a CTO. The Kaplan–Meier estimates for 10‐year survival were 26 (SE 5)% for patients with a CTO and 42 (SE 4)% for patients without a CTO, and the Kaplan–Meier estimates for other time points are shown in Table 4.

**Table 4:** Kaplan–Meier Estimates for Appropriate Device Therapy and All‐Cause Mortality Rates at Several Time Points

	Appropriate Device Therapy			All‐Cause Mortality		
	CTO (n=240)	No‐CTO (n=482)	*P* Value	CTO (n=240)	No‐CTO (n=482)	*P* Value
1‐y follow‐up	15.8%	11.2%	0.103	7.4%	7.3%	0.982
3‐y follow‐up	36.0%	22.6%	0.003	25.1%	16.5%	0.020
5‐y follow‐up	46.8%	32.8%	0.002	41.9%	27.8%	0.001

CTO indicates chronic total coronary occlusion.

On long‐term follow‐up, corrected for baseline patient and angiographic characteristics such as LVEF, the presence of a CTO was an independent predictor for appropriate device therapy (hazard ratio 1.394; 95% confidence interval, 1.060–1.832; 0.018) and there was a trend for higher all‐cause mortality (hazard ratio 1.269; 95% confidence interval, 0.996–1.616; *P*=0.054) (Tables 5 and 6).

**Table 5:** Influence of Patient and Angiographic Characteristics on All‐Cause Mortality

Characteristic	Univariate			Multivariate		
	HR	95% CI	*P* Value	HR	95% CI	*P* Value
Age	1.052	1.038–1.065	<0.001	1.050	1.036–1.064	<0.001
Male sex	0.878	0.634–1.215	0.433	···		
Hypertension	1.124	0.883–1.429	0.342	···		
Hypercholesterolemia	1.126	0.871–1.454	0.365	···		
Diabetes mellitus	1.682	1.309–2.162	<0.001	1.573	1.222–2.025	<0.001
Creatinine clearance	1.000	1.000–1.000	0.794	···		
QRS duration	1.001	1.000–1.001	<0.001	1.001	1.000–1.001	<0.001
LVEF	0.980	0.970–0.989	<0.001	0.982	0.972–0.991	<0.001
CTO	1.544	1.217–1.958	<0.001	1.269	0.996–1.616	0.054
Primary prevention	0.853	0.662–1.099	0.218	···		
MVD	1.899	1.395–2.586	<0.001	1.562	1.145–2.131	0.005

CI indicates confidence interval; CTO, chronic coronary total occlusion; HR, hazard ratio; LVEF, left ventricular ejection fraction; MVD, multivessel disease.

**Table 6:** Influence of Patient and Angiographic Characteristics on Appropriate Device Therapy

Characteristic	Univariate			Multivariate		
	HR	95% CI	*P* Value	HR	95% CI	*P* Value
Age	1.017	1.004–1.030	0.013	1.013	1.00–1.026	0.055
Male sex	1.901	1.200–3.011	0.006	1.757	1.107–2.787	0.017
Hypertension	1.051	0.800–1.380	0.723	···		
Hypercholesterolemia	0.930	0.701–1.234	0.616	···		
Diabetes mellitus	0.845	0.614–1.163	0.302	···		
Creatinine clearance	1.000	1.000–1.000	0.224	···		
QRS duration	1.000	1.000–1.001	0.302	···		
LVEF	1.000	0.997–1.002	0.750	0.999	0.989–1.008	0.801
CTO	1.529	1.166–2.005	0.002	1.394	1.060–1.832	0.018
Primary prevention	0.516	0.393–0.679	<0.001	0.548	0.416–0.723	<0.001
MVD	1.273	0.931–1.743	0.131	···		

CI indicates confidence interval; CTO, chronic coronary total occlusion; HR, hazard ratio; LVEF, left ventricular ejection fraction; MVD, multivessel disease.

### Influence of Primary Prevention Versus Secondary Prevention

**Figure 3:**
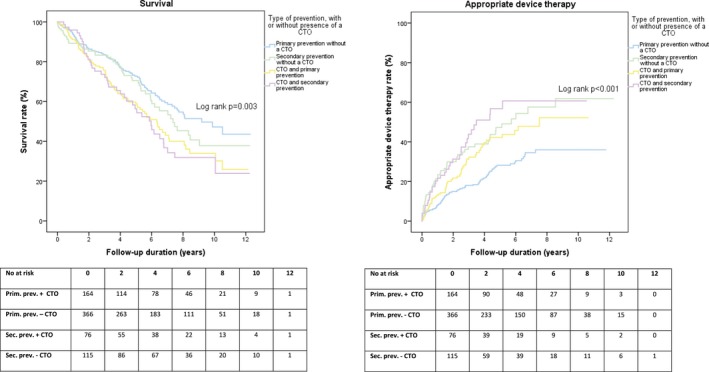
Influence on long‐term outcomes of the presence of a CTO in primary and secondary prevention. CTO indicates chronic total coronary occlusion.

Irrespective of whether the ICD was implanted for primary or secondary prevention, CTO patients had worse survival compared with non‐CTO peers. Primary prevention patients without a CTO experienced the lowest appropriate device therapy rate, compared with primary prevention patients with a CTO and secondary prevention patients (Figure 3).

### Current Available Data

**Figure 4:**
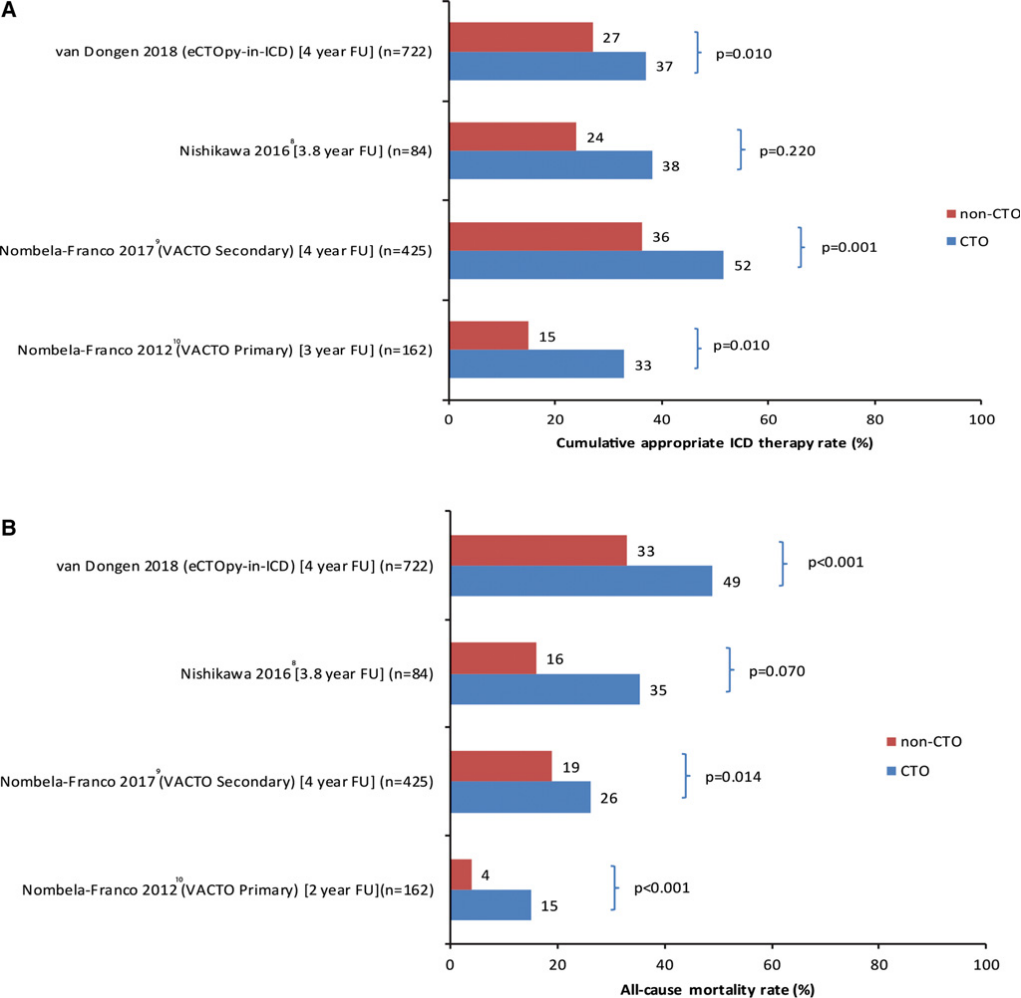
A, Overview of all‐cause mortality rates (%) per study. B, Overview of cumulative appropriate ICD therapy rates (%) per study. eCTOpy‐in‐ICD indicates evaluation of the impact of a Chronic Total coronary Occlusion on ventricular arrhythmias and long‐term mortality in patients with ischemic cardiomyopathy and an Implantable Cardioverter‐Defibrillator (this study); FU, follow‐up; ICD, implantable cardioverter defibrillator; VACTO, impact of chronic total coronary occlusion on recurrence of ventricular arrhythmias in ischemic secondary prevention implantable cardioverter‐defibrillator recipients (study).

In Figure 4A and 4B, an overview of the available event rates for all‐cause death and appropriate device therapy of all studies on this subject is depicted.^8^, ^9^, ^10^ Event rates were numerically higher in CTO patients with an ICD for ICM, compared with patients without a CTO.

### Influence of SVD, MVD, and CTO

**Figure 5:**
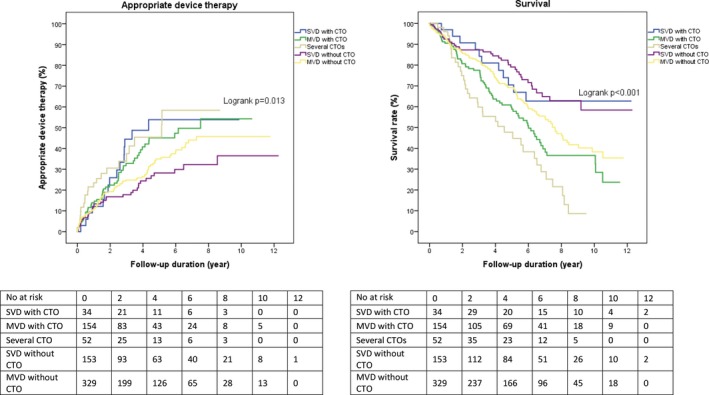
Influence on long‐term outcomes of single‐vessel disease, multivessel disease, and presence of a CTO. CTO indicates chronic total coronary occlusion; MVD, multivessel disease; SVD, single‐vessel disease.

Patients with SVD without a CTO experienced the lowest rate of appropriate device therapy over time, followed by patients with MVD without a CTO, compared with patients with a CTO (Figure 5). Furthermore, patients with SVD with or without a CTO had the highest survival rate compared with patients with MVD and a CTO (Figure 5).

### Influence of Collaterals

**Figure 6:**
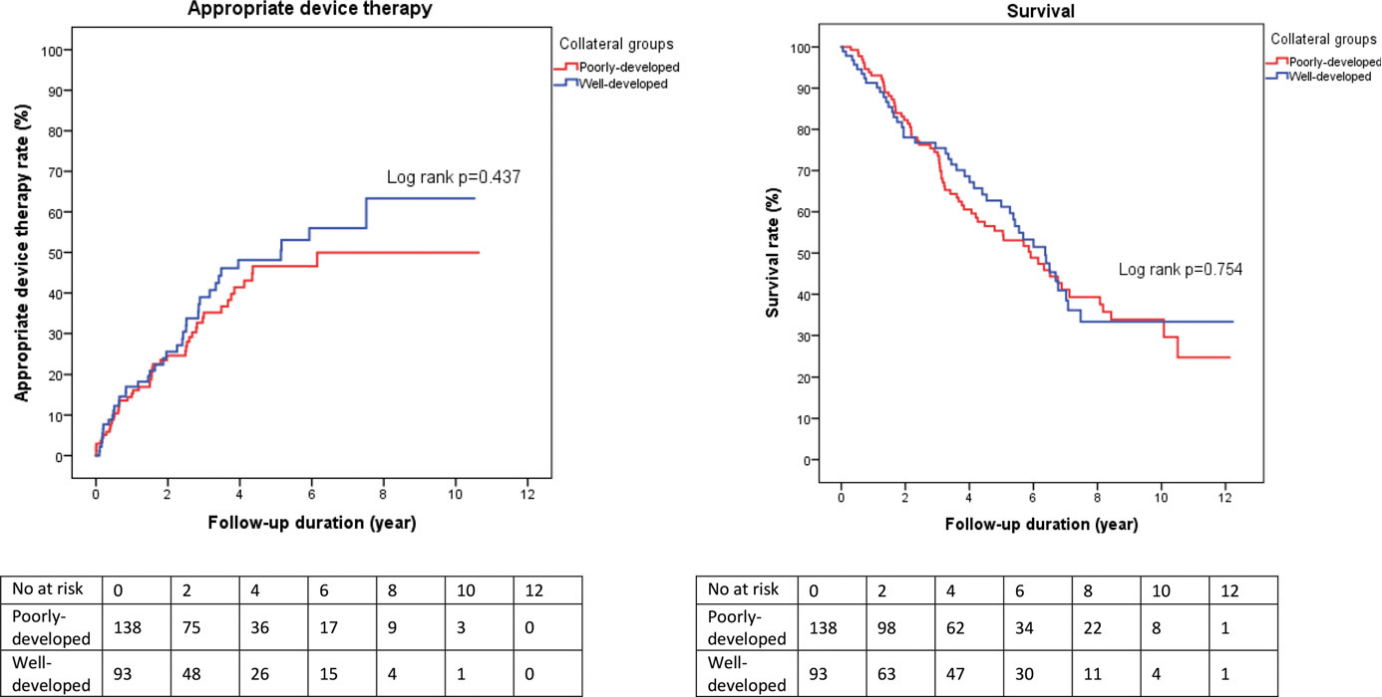
Influence on long‐term outcomes of poorly developed vs well‐developed collaterals.

In patients with a CTO, the presence of well‐developed collaterals did not seem to influence long‐term survival nor appropriate device therapy compared with patients with poorly developed collaterals (Figure 6).

### CTO Revascularization

**Figure 7:**
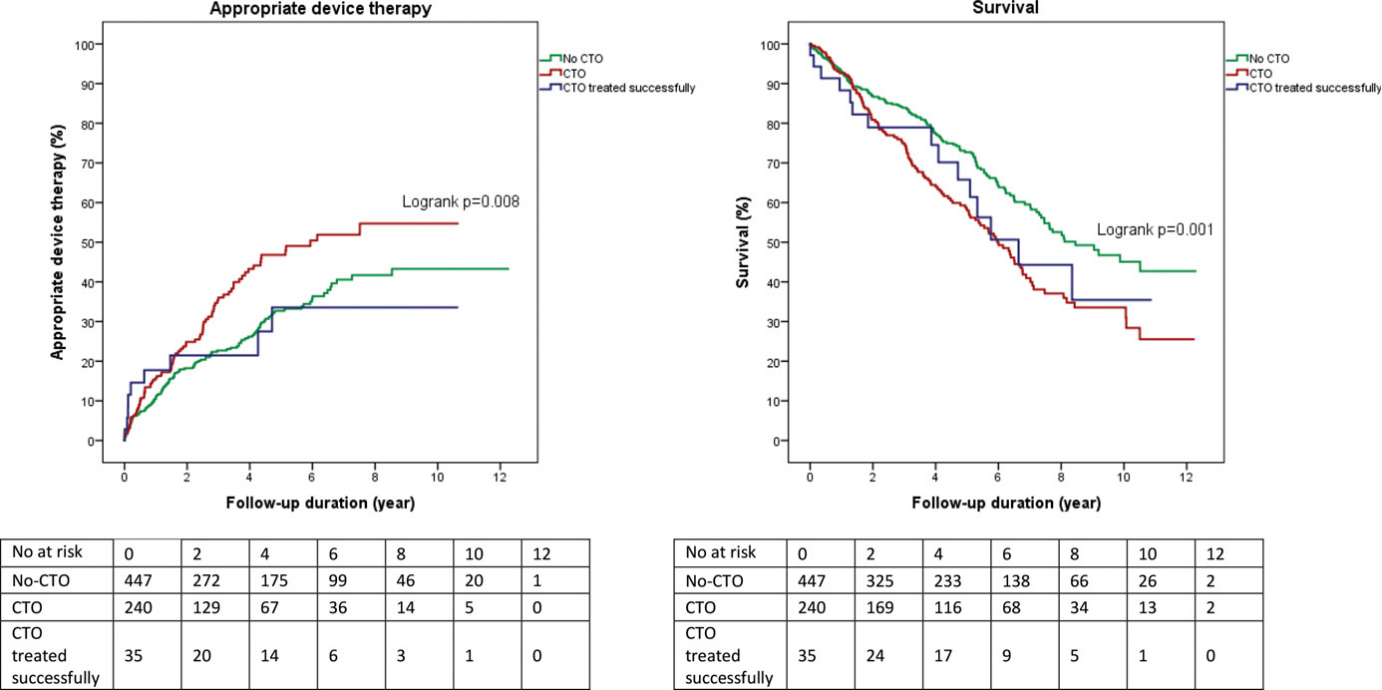
Influence on long‐term outcomes of CTO revascularization. CTO indicates chronic total coronary occlusion.

A small proportion of the CTO patients in this cohort was revascularized before the ICD implantation (n=35), mainly because of myocardial viability, inducible arrhythmias in the CTO territory, or severe CAD for which CABG was indicated. Patients with a revascularized CTO had similar appropriate device therapy rates compared with patients without a CTO (n=447) (Figure 7). Regarding long‐term survival, patients with a revascularized CTO had similar survival rates compared with patients with a CTO (Figure 7).

### Location of the CTO Lesion

**Figure 8:**
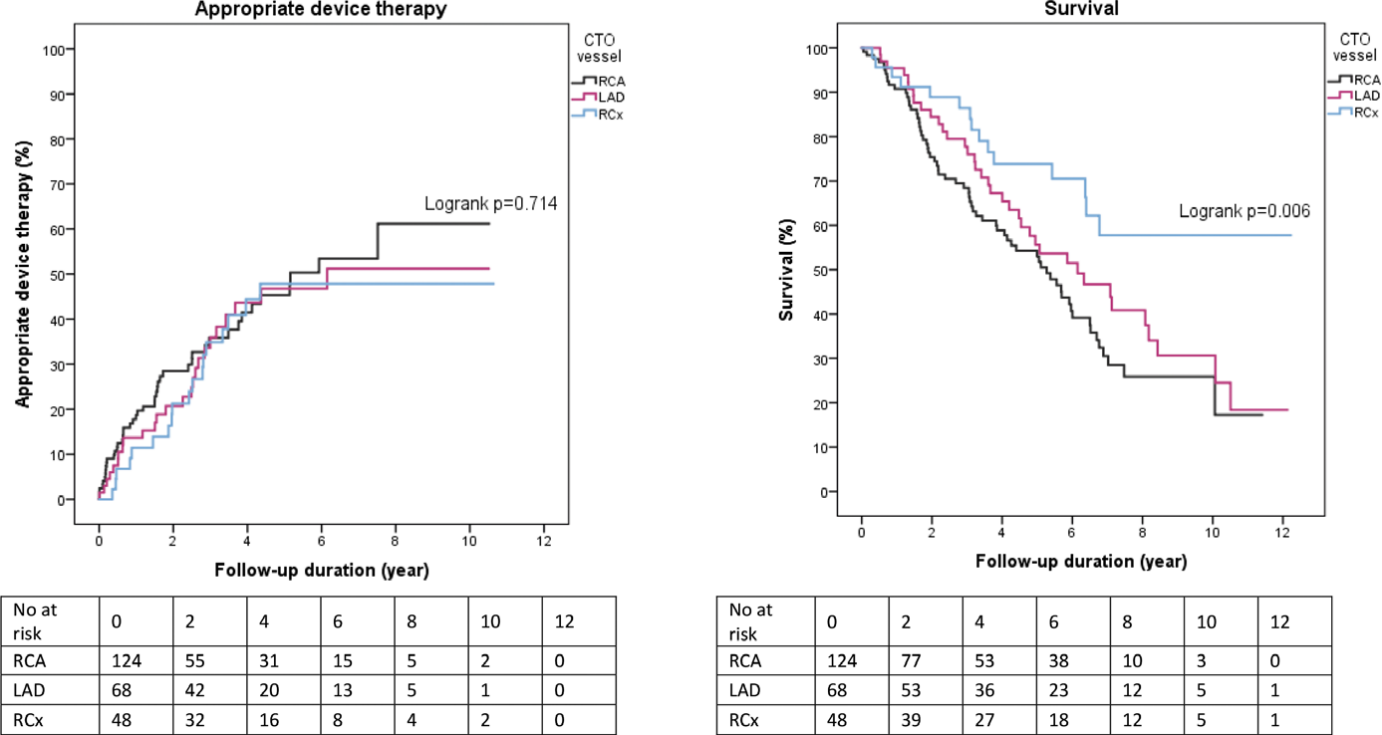
Influence of location of the CTO lesion. CTO indicates chronic total coronary occlusion; LAD, left anterior descending coronary artery; RCA, right coronary artery; RCx, ramus circumflexus coronary artery.

Appropriate device therapy rates were similar between patients with a CTO in the right coronary artery, left anterior descending coronary artery, or ramus circumflexus coronary artery (Figure 8). Survival was highest in patients with a CTO in the ramus circumflexus coronary artery (Figure 8).

## Discussion

The current study showed that on long‐term follow‐up, patients with an ICD for ischemic heart disease who also have a CTO (1) received more appropriate device therapy and (2) had a higher all‐cause mortality, compared with ICD patients with ICM but without a CTO; (3) corrected for several baseline characteristics (including age, LVEF, and QRS duration). The presence of a CTO was an independent predictor for more appropriate device therapy in this specific patient population.

### Currently Available Data

Previous published studies^8^, ^9^, ^10^, ^16^, ^17^ have shown a trend towards higher appropriate device therapy and all‐cause mortality rates in ICD patients with a CTO. Also, the presence of a CTO has proven to be associated with a worse survival compared with patients with SVD or MVD without a CTO.^3^, ^4^ The current, substantially larger, study confirms this observation. Regarding appropriate device therapy, Nombela‐Franco et al found that patients with an ICD for ICM and a CTO experience more device therapy both in primary (n=162) and secondary (n=425) prevention.^9^, ^10^ We have also observed this in our cohort, suggesting that the presence of a CTO has more impact on the myocardium than 1 or more narrowed coronary arteries.

In the COMMIT‐HF (Contemporary Modalities in Treatment of Heart Failure) substudy with patients with ischemic systolic heart failure (n=675), the presence of a CTO (n=278) was associated with an increased 2‐year all‐cause mortality compared with patients without a CTO (n=397). Also, 1‐year cardiovascular mortality and 1‐year major adverse cardiovascular event rates were significantly higher in the group of patients with a CTO,^6^ and (corrected for LVEF) the presence of a CTO was independently associated with a higher 1‐year mortality.

The cause of the high death rate in CTO patients, despite the presence of an ICD, remains unclear. In our cohort this does not appear to be driven by a higher occurrence of, for example, untreatable VAs in the patients with a CTO. It could be postulated that an ICD does decrease mortality from VAs, but that CTO patients have a higher mortality because of the presence of other comorbidities, such as diabetes mellitus. In addition, when looking at the MADITII risk score (which was developed in a primary prevention population post myocardial infarction) in our population,^15^ a trend towards less CTO patients with risk score 0 and more CTO patients with risk scores of 1 and 2 can be appreciated. In the MADITII population,^15^ these risk score groups were at lower risk for mortality. Furthermore, in a large portion of all deaths in our cohort, the exact cause was unknown. Hypothetically, these CTO patients could have died more frequently from arrhythmic causes untreatable by an ICD, which would be unknown since their deaths would most likely have been ruled as from natural causes and the ICD would not have been read out postmortem.

The pathophysiology of VAs is important and accordingly, the cause of the CTO could be just as important. Di Marco et al have shown that the presence of a CTO in a previous infarcted area (infarct‐related artery [IRA]‐CTO) is associated with the occurrence of more VA (especially fast VT/VF) and more ICD therapy.^18^, ^19^ VT ablation could be an appropriate treatment option in these patients. Nevertheless, Di Marco et al have also shown that the presence of an infarct‐related artery–CTO is an independent predictor of VT recurrence after VT ablation, compared with patients with prior myocardial infarction without an infarct‐related artery–CTO.^20^

Most CTOs are infarct‐related artery–CTOs,^1^ so this could mean that in CTO patients most likely a larger infarct zone is present, leading to more VTs. Combined with a reduced ischemia reserve (during stress, exercise), leading to more VF, the CTO could be a strong substrate for more arrhythmias. In the eCTOpy‐in‐ICD population more appropriate therapy was observed in the CTO group, because of both more VT and more VF, compared with the non‐CTO group.

### Collaterals

The influence of collateral vessels to a CTO has been investigated sparsely. One registry showed that the presence of well‐developed collaterals to a concomitant CTO in ST‐segment elevation myocardial infarction patients was associated with better long‐term survival.^21^ In our cohort of stable ICD patients, we found no clear effect of the quality of collateral vessels on survival or on appropriate device therapy, although appropriate device therapy appeared to occur more frequently in patients with well‐developed collaterals. In the VACTO secondary (impact of chronic total coronary occlusion on recurrence of ventricular arrhythmias in ischemic secondary prevention implantable cardioverter‐defibrillator recipients) study, a trend towards more appropriate therapy was observed in patients with Rentrop 3 collaterals.^9^ The authors hypothesize that hibernating myocardium may explain this finding.^9^ In patients with good collateral filling of the CTO vessel, more viability could be present, especially in a border zone around the necrotic core of the area affected by the CTO.^9^ This hypothesis has been underscored in a cardiac magnetic resonance imaging study of patients in the EXPLORE (Evaluating xience and left ventricular function in PCI on occlusions after STEMI) trial (ST‐segment elevation myocardial infarction patients with a concurrent CTO [n=302]). Patients with well‐developed collaterals showed an improved restoration of dysfunctional segments in the CTO territory, compared with patients with poorly developed collaterals.^22^ In addition, Werner et al showed that few patients with well‐developed collaterals show a normal coronary flow reserve, which suggests that even in these patients episodes of ischemia can occur, which would be a trigger for VA.^9^, ^23^

### Primary Versus Secondary Prevention in Combination With a CTO

The presence of a CTO results in higher all‐cause mortality rates in patients with an ICD for ICM for both primary and secondary prevention. The presence of a CTO has, however, a less abundant effect on appropriate device therapy in secondary prevention patients in the long term. However, the relatively lower number of secondary prevention patients in our cohort (n=191) could have played a role in this observed difference. On the Kaplan–Meier curve, at 2‐year follow‐up a clear divergence of the appropriate therapy rates in the secondary prevention patients with a CTO can be observed. This coincides with the observations made in the VACTO secondary study. In their population (n=425) of secondary prevention patients, the presence of a CTO was associated with, and was an independent predictor for, both decreased survival and more appropriate therapy.^9^

### Treatment of a CTO

Since it has been established that patients with a CTO experience more VAs and have a lower life expectancy, one of the most significant questions is whether CTO revascularization can improve outcomes. In general registry populations of patients with a CTO, CTO revascularization seems to improve outcomes.^1^, ^24^ Regarding patients with an ICD for ICM and a CTO, little is known about the effect of revascularization. Raja et al compared patient groups without a CTO, with a CTO, and with a revascularized CTO. Their analyses did not show any statistical differences between the 3 groups.^16^ Yap et al looked at outcomes in a small subgroup (n=25) of out of hospital cardiac arrest patients with a CTO, and found that compared with patients with an unrevascularized CTO, event rates were similar. The current study showed that appropriate device therapy rates in patients undergoing CTO revascularization (n=35) before ICD implantation were similar to the rates found in the non‐CTO group (n=447). The currently limited available data is thus conflicting with regard to the question of whether CTO revascularization influences the increased occurrence of VAs in CTO patients, but our data might suggest that CTO revascularization could improve electrical stability and reduce appropriate device therapy, which is a heavy burden for ICD patients. In addition, one could question the need for an ICD in patients with a successfully revascularized CTO.

In a systematic review and meta‐analysis of studies investigating the effect of CTO revascularization on ECG parameters, we found that directly after successful revascularization, several ECG parameters improved compared with before CTO revascularization.^7^ However, in most of the included studies in this review, no clinical follow‐up data were available.^7^ Therefore, whether CTO revascularization truly ameliorates ventricular arrhythmogenicity remains unclear. Furthermore, what type of CTO revascularization should be preferred (CTO, PCI, or CABG) is also unknown.

### Limitations

This study has several limitations that should be taken into account when interpreting the data. First, this study is a registry study, with all associated limitations. Importantly, because of the retrospective nature of this study, there is a lack of insight into reasons whether or not to revascularize the diagnosed CTOs. Also, in some of the patients the exact cause of death could no longer be determined, and since in The Netherlands it is not customary to read out the ICD postmortem, no information on arrhythmias premortem was available. Second, for some subanalyses the numbers are low, so these analyses should be considered purely as hypothesis generating.

## Conclusion

The current study shows that in a large cohort of patients with an ICD for ICM, the presence of a CTO is associated with more appropriate device therapy and worse survival at long‐term follow‐up, compared with patients without a CTO. Moreover, the presence of a CTO in patients with an ICD is an independent predictor for the occurrence of VAs leading to more appropriate device therapy. CTO revascularization might ameliorate this negative effect of a CTO on the myocardium.

## Disclosures

None.
